# CD24 staining of mouse mammary gland cells defines luminal epithelial, myoepithelial/basal and non-epithelial cells

**DOI:** 10.1186/bcr1371

**Published:** 2005-12-12

**Authors:** Katherine E Sleeman, Howard Kendrick, Alan Ashworth, Clare M Isacke, Matthew J Smalley

**Affiliations:** 1 Breakthrough Breast Cancer Research Centre, The Institute of Cancer Research, Fulham Road, London SW3 6JB, UK

## Abstract

**Introduction:**

Breast cancer is thought to arise in mammary epithelial stem cells. There is, therefore, a large amount of interest in identifying these cells. The breast is a complex tissue consisting of two epithelial layers (an outer myoepithelial/basal layer and an inner luminal epithelial layer) as well as a large non-epithelial component (fibroblasts, endothelial cells, lymphocytes, adipocytes, neurons and myocytes). The definitive identification of a mammary epithelial stem cell population is critically dependent on its purity. To date, this has been hampered by the lack of suitable markers to separate out the two epithelial layers, and to remove contaminating non-epithelial cells.

**Methods:**

Mouse mammary glands were dissociated and stained with CD24. Cells were sorted into separate populations based on CD24 expression and assessed for luminal epithelial and myoepithelial/basal markers by direct fluorescent microscopy and real time PCR. The stem/progenitor potential of these cell populations was assessed *in vivo *by cleared mammary fat pad transplantation.

**Results:**

Three populations of CD24 expressing cells were identified: CD24^Negative^, CD24^Low ^and CD24^High^. Staining of these cells with cytokeratin markers revealed that these populations correspond to non-epithelial, myoepithelial/basal and luminal epithelial cells, respectively. Cell identities were confirmed by quantitative PCR. Cleared mammary fat pad transplantation of these cell populations revealed that extensive mammary fat pad repopulation capacity segregates with the CD24^Low ^cells, whilst CD24^High ^cells have limited repopulation capacity.

**Conclusion:**

Differential staining of mammary epithelial cells for CD24 can be used to simultaneously isolate pure populations of non-epithelial, myoepithelial/basal and luminal epithelial cells. Furthermore, mammary fat pad repopulation capacity is enriched in the CD24^Low ^population. As separation is achieved using a single marker, it will be possible to incorporate additional markers to further subdivide these populations. This will considerably facilitate the further analysis of mammary epithelial subpopulations, whilst ensuring high purity, which is key for understanding mammary epithelial stem cells in normal tissue biology and carcinogenesis.

## Introduction

There is increasing evidence that normal tissue stem cells are the cells of origin of many cancers, and the identification of such cells is, therefore, key to understanding the aetiology of carcinogenesis [1-4]. Stem cell identification strategies rely on the prospective isolation of candidate cell populations using cell surface markers, followed by *in vivo *functional assays in mouse models [5-7]. The accurate definition and characterisation of a potential stem cell population, however, is dependent on its purity. The normal breast is a heterogeneous tissue, consisting of two epithelial layers, an inner luminal epithelial layer and an outer myoepithelial/basal layer, as well as non-epithelial cells. The enormous epithelial proliferation, differentiation and regression that occurs with each pregnancy provides indirect evidence for the existence of a mammary epithelial stem cell. Transplant data suggest three different mammary stem cell compartments may exist [6] and candidate cell types have been suggested (reviewed in [4]). Histological studies have suggested that stem cells within the mammary epithelium are likely to reside in a suprabasal location [4]. However, the direct identification of mammary gland stem cells remains elusive. This is predominantly due to the paucity of appropriate markers for the separation of the luminal epithelial and myoepithelial/basal cell populations into their component subpopulations. Such separation is necessary in order to accurately assay populations of putative mammary stem cells by cleared fat pad transplantation.

CD24 has generated recent interest as a potential marker of human breast cancer stem cells [8]. To explore the potential of this marker for isolating subpopulations of normal epithelial cells from the adult virgin mouse mammary gland, we used flow cytometry to investigate CD24 expression in mouse mammary cell preparations. Staining with CD24 revealed three distinct cell populations: CD24^High^, CD24^Low ^and CD24^Negative^. Analysis of cytoskeletal antigen staining and of gene expression patterns demonstrated that these populations represented luminal epithelial, myoepithelial and non-epithelial cells, respectively. Mammary fat pad repopulation assays revealed that the CD24^Low ^population is enriched for stem/progenitor activity.

CD24-based separation of mouse mammary luminal epithelial and myoepithelial cell populations represents a major advance towards the prospective isolation of mammary stem cells.

## Materials and methods

### Preparation of single mammary cell suspensions

The fourth mammary fat pads were harvested from 20 virgin female 10 to 12 week old FVB mice following removal of the intra-mammary lymph nodes. Fat pads were chopped three times with a McIlwain Tissue Chopper (Mickle Laboratory Engineering Company, Gomshall, Surrey, UK) set to cut at 100 μm intervals and the finely minced tissue was transferred to a digestion mix consisting of serum-free Leibowitz L15 medium (Sigma, Poole, Dorset, UK) containing 3 mg/ml collagenase A (Sigma) and 1.5 mg/ml trypsin (Sigma). This was incubated for 1 h at 37°C in a shaking incubator to liberate a mixture of epithelial tissue fragments ('organoids'), non-epithelial fragments (such as pieces of blood vessel or nerve bundles) and non-epithelial single cells. These were washed by pelleting and resuspension in L15/10% FCS to remove the collagenase mix and fatty waste, and then incubated with red blood cell lysis buffer (Sigma) to remove erythrocytes (2 × 5 minutes). Following a further wash in L15/10% FCS, the sample was 'pre-plated' in Dulbecco's modified Eagle's medium (Invitrogen, Paisley, UK) with 10% FCS (Sigma) for 1 h at 37°C/5% CO_2_. The majority of the contaminating fibroblasts, which are single cells, attach to the tissue culture plastic in this time, whereas the epithelial organoids do not. The epithelial and non-epithelial fragments, and contaminating single cells such as lymphocytes and some fibroblasts, can then be poured off, leaving the majority of the fibroblasts behind.

To obtain single mammary epithelial cells, the organoid preparations were washed twice in Ca^2+^/Mg^2+^-free PBS/0.02% w/v EDTA and incubated for 15 minutes in Joklik's Modification of Minimal Essential Medium for Suspension Culture (Sigma), to allow cell-cell contacts to begin to break down. The sample was then pelleted and resuspended in 2 ml 0.25% w/v trypsin/0.2% w/v EDTA in Hank's Balanced Salt Solution (Sigma). This was incubated at 37°C for 2 minutes. Release of DNA from damaged cells at this stage causes clumping, so after the 2 minute incubation a P1000 pipette was used to disaggregate DNA clumps and then 5 ml of serum-free L15 medium containing 1 μg/ml type I DNase (Sigma) was added. The sample was incubated for a further 5 minutes at 37°C and then an equal volume of L15/10% FCS added to stop the trypsinisation. Remaining clumps were removed by filtration through a 40 μm cell strainer and the resultant single cells pelleted, resuspended in fresh L15/10% FCS and counted.

### Flow cytometry and cell sorting

Cells were incubated at 10^6^/ml in L15/10% FCS with anti-CD24-fluorescein isothiocyanate (clone M1/69, BD Biosciences, Oxford, UK, 0.5 μg/ml) and anti-CD45-phycoerythrin-Cy5 (clone 30-F11, BD Biosciences, Oxford, UK, 0.25 μg/ml) for 45 minutes at 4°C. Cells were washed in L15/10% FCS and resuspended in L15/10% FCS/0.01% 4',6-diamidino-2-phenylindole dihydrochloride (DAPI).

Analysis was carried out on a BD FACSVantageSE DiVa (BD Biosciences) equipped with two Coherent 90 C-4 argon ion lasers (Coherent, Santa Clara, CA, USA) set at 488 nm and 333.6 to 333.8 nm. Samples were gated on the basis of forward- and side-scatter. Dead cells (DAPI bright) and leukocytes (CD45^+^) were excluded. Doublets and higher order clumps were excluded using a time-of-flight approach, where forward-scatter-height was plotted against forward-scatter-area. Routine examination of sorted cells revealed >99% single cellularity.

### Confocal immunofluorescent staining

Cells were sorted directly onto poly-L-lysine coated slides, air dried, and stored at -20°C. The cells were fixed in 1:1 methanol acetone at -20°C for 5 minutes and stained with antibodies against cytokeratin (CK)14 (clone LL002, Lab Vision, Suffolk, UK, 2.1 μg/ml) or CK8/18 (clone 5D3, Novocastra, Vision Biosystems, Newcastle-upon-Tyne, UK, 2 μg/ml) in addition to a nuclear stain (DAPI). Secondary antibodies were isotype specific goat anti-mouse antibodies (A21127, A21157, A21126; Invitrogen, Paisley, UK). Lack of non-specific staining by secondary antibodies was confirmed using isotype matched control primary antibodies (clones 15H6 and B10, Southern Biotech, Birmingham, Alabama, USA). Lack of cross reactivity in double staining experiments was confirmed with controls incubated with single primary antibodies and both secondary antibodies.

### Quantitative PCR analysis

Quantitative real time reverse transcription PCR (qPCR) reactions were carried out to determine fold changes in expression of a selection of genes with known luminal epithelial (*Lactotransferrin *(*Ltf*), *Milk Fat Globule-EGF Factor 8 protein *(*Mfge8*), *CK18 *(*Krt1-18*)), myoepithelial/basal (*CK14 *(*Krt1-14*), *Myosin Light Polypeptide 6 *(*Myl6a*)) or non-epithelial (*Myl6a*, *Procollagen 3a1 *(*Col3a1*), *CD31*) distribution, compared to leucocyte-depleted, bulk mammary cells. Populations were freshly sorted into tubes rinsed with FCS, resuspended in Trizol reagent (Invitrogen, Paisley, UK), and stored at -20°C. RNA was extracted according to the manufacturer's instructions. cDNA synthesis was carried out using Sensiscript RT kit (Qiagen, Crawley, UK). Up to 50 ng of RNA was transcribed into cDNA using an oligo dT^n ^primer (Promega, Southampton, UK) per reaction; 1 μl of cDNA was used per qPCR reaction. Each analysis reaction was performed in triplicate. GAPDH was used as an endogenous control throughout all experimental analyses. Gene expression analysis was performed using TaqMan Gene Expression Assays on an ABI Prism 7900HT sequence detection system (Applied Biosystems, Foster City, California, USA). Analysis was performed using the Δ-ΔCt method, which determines fold changes in gene expression relative to a comparator sample (mammary epithelial cells that had been depleted for CD45^+ ^cells but not separated further). Significant deviation of the mean value of each sample group from a fold difference of 1 (no change compared to the comparator sample) was tested using a t-test on Log_10 _transformed data. Standard curves were derived from sequence verified cDNA IMAGE clones (MRC Geneservice, Cambridge, UK) for each gene of interest confirming that sample amplification was within the linear range of the assay (data not shown).

### Cleared mammary fat pad transplantation

Freshly isolated cells were low pressure sorted into sterile screw-cap Eppendorf tubes and pelleted in an Eppendorf benchtop microfuge at 700 × *g *for 5 minutes. Cell pellets were resuspended in fresh PBS, counted, and resuspended in serum-free L15 medium at an appropriate cell density (such that 10 μl of serum-free L15 contained the number of cells to be implanted in each fat pad). There was no intervening culture period prior to transplantation.

Transplantation into cleared fourth mammary fat pads of 21-day old syngeneic female mice was carried out as described [5]. All animal work was approved by Local Ethics Committee and carried out under Home Office approval. Eight weeks after transplantation, fat pads were wholemounted and analysed as described [5]. They were scored as negative for outgrowth if no epithelial structures could be observed. They were scored as 'failed clears' if they contained an epithelial ductal network that could be seen to have grown in from one edge of the fat pad and in which the majority of ductal branching had the same directionality. If outgrowths could be seen to have originated from a central region of the cleared fat pad and the directionality of the ductal branching was different in different parts of the fat pad, they were scored as successful transplants. Successful transplants had a region of epithelial outgrowth dissected out under a binocular microscope for paraffin embedding by routine methods and routine immunocytochemistry to detect α-isoform smooth muscle actin (clone 1A4; Sigma).

## Results

**Figure 1:**
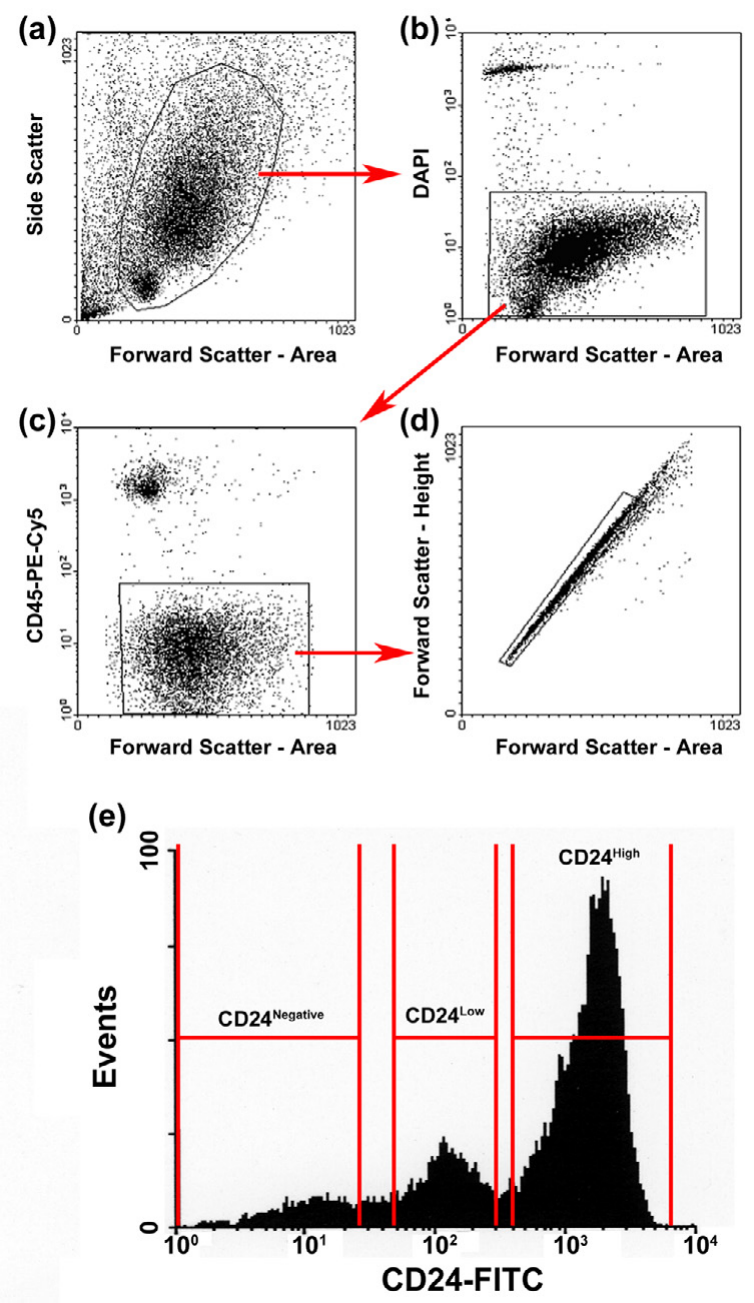
Differential CD24 expression distinguishes between mammary gland subtypes. (**a**) Cells were sorted on the basis of forward scatter and side scatter. (**b**) Dead cells (DAPI bright) were excluded. (**c**) B lymphocytes and immature T lymphocytes are CD24^+ ^and, therefore, CD45 staining was used to exclude all leukocytes. (**d**) Doublets and higher order cell clumps were excluded using a time-of-flight approach (boxed cells represent single cells). (**e**) Typical CD24 staining profile, indicating the three populations of cells: CD24^High ^(69.9 ± 5.5%; n = 15), CD24^Low ^(22.2 ± 5.6%; n = 15) and CD24^Negative ^(6.3 ± 1.5%; n = 15). DAPI = 4',6-diamidino-2-phenylindole dihydrochloride; FITC, fluorescein isothiocyanate; PE-Cy5, phycoerythrin-Cy5.

**Figure 2:**
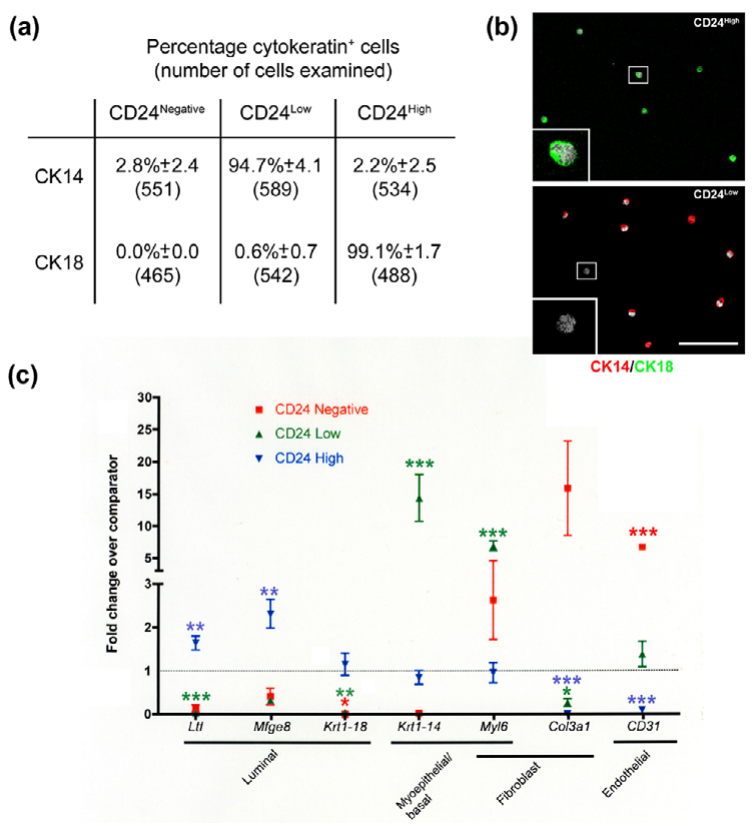
Characterisation of CD24^High^, CD24^Low ^and CD24^Negative ^populations. (**a**) Cell populations sorted on the basis of CD24 expression were stained for cytokeratin (CK)8/18 or CK14. The mean percentage of CK14 and CK8/18 positive cells (±standard deviation) and the total number of cells counted is indicated for each population. Results from four independent sorts. (**b**) Cells were double stained with CK14 and CK8/18. Only CK18^+^/CK14^- ^cells were observed in the CD24^High ^population (top panel). The majority of CD24^Low ^cells were CK14^+^, with occasional CK18^-^/CK14^- ^cells (bottom panel, inset). No CK18^+^/CK14^+ ^cells were observed. Scale bar = 75 μm. (**c**) Quantitative rtPCR reactions were carried out to determine fold changes in expression of a selection of genes with known luminal epithelial (*Ltf*, *Mfge8*, *Krt1-18*), myoepithelial/basal (*Krt1-14*, *Myl6a*) or non-epithelial (*Myl6a*, *Procollagen 3a1 *(*Col3a1*), *CD31*) distribution, compared to leucocyte-depleted, bulk mammary cells. The analysis was carried out on two independent cDNA syntheses from each of three independent sorts (CD24^Low ^and CD24^High^) or on two independent cDNA syntheses from one sort and a single cDNA synthesis from a pool of two further sorts (CD24^Negative^). Significance levels are indicated by: *P < 0.05; **P < 0.01; ***P < 0.001.

Staining with CD24 consistently revealed three distinct populations: CD24^High ^(69.9 ± 5.5%), CD24^Low ^(22.2 ± 5.6%), and CD24^Negative ^(6.3 ± 1.5%) (results from 15 independent sorts; Fig. 1). To examine the phenotype of these populations, cells were sorted directly onto poly-L-lysine coated slides and stained with antibodies against CK8/18, expressed exclusively by luminal epithelial cells *in vivo*, or CK14, expressed exclusively by myoepithelial/basal cells *in vivo*. Of the CD24^High ^population, 99.1 ± 1.7% (n = 488 cells from 4 independent sorts) were CK8/18^+ ^luminal cells. Of the CD24^Low ^population, 94.7 ± 4.1% (n = 589 cells from 4 independent sorts) were CK14^+ ^myoepithelial/basal cells. The majority of CD24^Negative ^cells were both CK8/18 negative and CK14 negative, indicating that this population comprised mainly non-epithelial cell types (Fig. 2a). Double staining of cells for both CK8/18 and CK14 (Fig. 2b) revealed the presence of CK negative cells within the CD24^Low ^population (bottom panel, inset), but failed to identify cells in any population that were both CK8/18^+ ^and CK14^+ ^(data not shown).

To independently assess the luminal epithelial, myoepithelial/basal and non-epithelial nature of the CD24^High^, CD24^Low ^and CD24^Negative ^cells, respectively, qPCR for a selection of genes was undertaken. Genes associated with luminal epithelial cell function (*Ltf *and *Mfge8*) were significantly (*p* < 0.01) enriched in the CD24^High ^population. Genes associated with myoepithelial/basal cells (*Krt1-14*, *Myl6*) were similarly enriched in the CD24^Low ^cells (*p* < 0.001). Expression of *CD31 *was significantly (*p* < 0.001) elevated in the CD24^Negative ^population, confirming that this population was non-epithelial (Fig. 2c and Additional file 1; results from three independent sorts).

**Figure 3:**
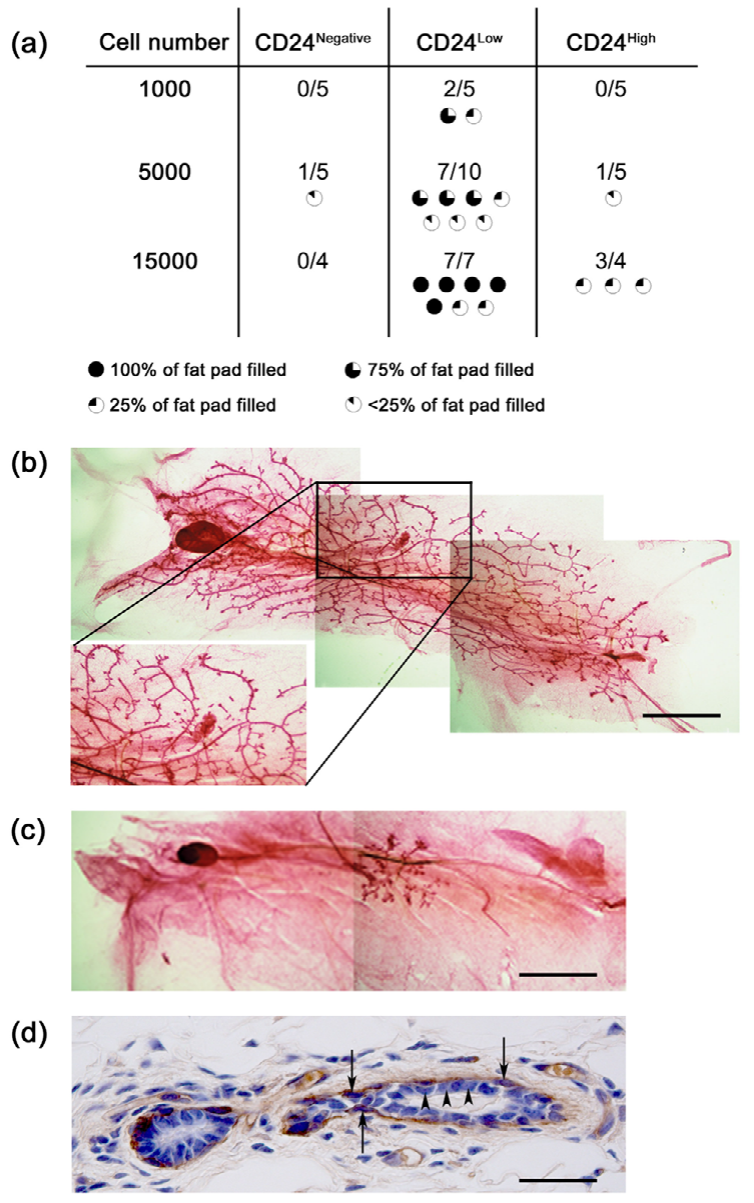
Transplantation of CD24^High^, CD24^Low ^and CD24^Negative ^populations. Cell populations sorted on the basis of CD24 expression were transplanted into cleared mammary fat pads of syngeneic FVB mice. (**a**) Table indicates the number of cells of each population injected into each fat pad, the total number of fat pads injected, and the number of transplants that generated epithelial outgrowths. Failed clears were excluded from the analysis. The extent to which each outgrowth filled the host fat pad is indicated graphically. CD24^Low ^cells showed the highest rate of successful transplantation and formed the most extensive outgrowths at each cell number studied. (**b**) Example of an outgrowth derived from transplantation of CD24^Low ^cells that fills 100% of the host fat pad. The magnified boxed region highlights the point of origin of the outgrowth. Scale bar = 4.25 mm. (**c**) Example of an outgrowth derived from transplantation of CD24^High ^cells which fills <25% of the host fat pad. Scale bar = 4.25 mm. (**d**) Section through CD24^Low ^outgrowth stained with anti-α-isoform smooth muscle actin showing the positive outer myoepithelial layer (arrows) and the negative inner luminal epithelial layer (arrowheads). Scale bar = 40 μm.

To demonstrate the value of CD24 staining of mouse mammary cell populations for mammary epithelial stem cell discovery, cells from each of the three populations defined by CD24 staining were transplanted into the cleared mammary fat pads of syngeneic mice. CD24^Low ^cells had the greatest outgrowth potential, with 1,000 cells producing outgrowths in 2 of 5 cases (Fig. 3a). CD24^Low ^cells also had the most extensive mammary fat pad repopulating capacity, and could give rise to outgrowths that filled the entire fat pad (Fig. 3a,b) and consisted of both luminal epithelial and myoepithelial cell layers (Fig. 3d). CD24^High ^cells formed fewer outgrowths than CD24^Low ^cells and had a limited repopulating capacity, forming outgrowths that only filled up to 25% of the mammary fat pad, even when 15,000 cells were injected (Fig. 3a,c).

## Discussion

The primary goal of mammary epithelial separation strategies is to define a population of mammary epithelial stem cells for functional analysis. To date, two such strategies have been described: the use of Hoechst 33342 dye to define a side population [5,9], and the use of the cell surface marker Sca-1 [7]. There is some evidence that mammary epithelial cells lying within the side population are enriched for stem activity [5,10]. Analysis of these experiments is confounded, however, by the toxic effects of Hoechst 33342 on cell viability [11]. Furthermore, Bcrp1 (the pump responsible for the efflux of Hoechst 33342) is present and active in mature secretory luminal cells, indicating that it cannot be an exclusive stem cell marker [12]. Sca-1, a marker of haematopoietic stem cells, is expressed on 20% of mammary gland cells, and Sca-1^+ ^cells have been suggested to be enriched for stem activity [7]. However, a subset of stromal cells are Sca-1^+ ^[13] (M Smalley, data not shown), indicating that Sca-1^+ ^cells freshly isolated from the mammary gland are a heterogeneous mix of epithelial cells and fibroblasts.

The accurate identification of an epithelial stem cell subpopulation is critically dependent on its purity. Current methods for the isolation of luminal epithelial and myoepithelial cells from the mouse mammary gland rely on two separate rat monoclonal antibodies, 33A10 and JB6, respectively [14]. These antibodies are not available as fluorochrome conjugates; therefore, it has not been possible to isolate both of these epithelial populations whilst simultaneously avoiding non-epithelial cells. Furthermore, recent data indicate that 33A10 does not stain the total luminal epithelial cell population (M Smalley, manuscript in preparation). The data shown here demonstrate that differential staining of CD24 can be used for the simultaneous isolation of mouse mammary luminal epithelial, myoepithelial/basal and non-epithelial cells at high levels of purity. Furthermore, it has been shown that whilst a mammary epithelial progenitor cell with limited outgrowth potential is found within the CD24^High ^cells, extensive mammary fat pad repopulation ability segregates with the CD24^Low ^population. This is consistent with the balance of evidence, which suggests that stem cells reside in a suprabasal location [4]. The incorporation of multiple additional fluorochromes for flow cytometric separation will enable these stem/progenitor cells to be definitively isolated. Consequently, this method will prove indispensable for the functional analysis of mammary gland epithelial subpopulations, which is essential for understanding the role of stem cells in normal tissue biology and carcinogenesis.

## Conclusion

We describe a simple and efficient procedure for the simultaneous identification and isolation of luminal epithelial, myoepithelial/basal and non-epithelial cells from the mouse mammary gland, and show that extensive mammary fat pad repopulating ability segregates with the myoepithelial/basal population. CD24 staining of the mouse mammary epithelium has three significant advantages over any current mammary gland cell separation strategy. First, it provides a simple and efficient means of separating mouse mammary epithelial from non-epithelial cells. Second, it enables simultaneous identification and isolation of pure populations of luminal and myoepithelial cells. Third, as separation is achieved with a single marker, CD24 can be used in conjunction with multiple additional markers for the prospective isolation of mammary stem cells. This represents a major advance in the study of mouse mammary gland biology.

## Abbreviations

CK = cytokeratin; DAPI = 4',6-diamidino-2-phenylindole dihydrochloride; FCS = foetal calf serum; PBS = phosphate-buffered saline; qPCR = quantitative rtPCR.

## Competing interests

The authors declare that they have no competing interests.

## Authors' contributions

KS carried out the mammary epithelial cell harvest, cell sorting, cellular analyses, *in vivo *transplantation assays, participated in the project design, and wrote the manuscript. HK carried out the molecular analysis. AA, CMI and MS participated in the project design and helped to draft the manuscript. All authors read and approved the final manuscript.

## Supplementary Material

Additional file 1 A spreadsheet (XLS) table showing the results of qPCR analysis of gene expression within cell populations isolated by CD24 staining. Mean fold change over the comparator population (bulk CD45^- ^mammary cell preparations) and the range of fold change values are shown for each sample tested. Click here for file
